# Reporter Dyes Demonstrate Functional Expression of Multidrug Resistance Proteins in the Marine Flatworm *Macrostomum lignano*: The Sponge-Derived Dye Ageladine A Is Not a Substrate of These Transporters

**DOI:** 10.3390/md11103951

**Published:** 2013-10-16

**Authors:** Kristin Tietje, Georgina Rivera-Ingraham, Charlotte Petters, Doris Abele, Ralf Dringen, Ulf Bickmeyer

**Affiliations:** 1 Carl von Ossietzky University Oldenburg, Carl von Ossietzky Str. 9-11, Oldenburg D-26111, Germany; E-Mail: Kristin.Tietje@uni-oldenburg.de; 2 Alfred-Wegener-Institut Helmholtz-Zentrum für Polar- und Meeresforschung, Am Handelshafen 12, Bremerhaven D-27570, Germany; E-Mails: Georgina.Rivera-Ingraham@awi.de (G.R.-I.); Doris.Abele@awi.de (D.A.); 3 Centre for Biomolecular Interactions Bremen, University of Bremen, P.O. Box 33 04 40, Bremen D-28334, Germany; E-Mails: petters@uni-bremen.de (C.P.); ralf.dringen@uni-bremen.de (R.D.)

**Keywords:** 2-photon laser scanning microscopy, natural product, fluorescence, live imaging, ABC transporter

## Abstract

The marine plathyhelminth *Macrostomum lignano* was recently isolated from Adriatic shore sediments where it experiences a wide variety of environmental challenges, ranging from hypoxia and reoxygenation, feeding on toxic algae, to exposure to anthropogenic contaminants. As multidrug resistance transporters constitute the first line of defense against toxins and toxicants we have studied the presence of such transporters in *M. lignano* in living animals by applying optical methods and pharmacological inhibitors that had been developed for mammalian cells. Application of the MDR1 inhibitor Verapamil or of the MRP1 inhibitors MK571 or Probenecid increased the intracellular fluorescence of the reporter dyes Fura-2 am, Calcein am, Fluo-3 am in the worms, but did not affect their staining with the dyes Rhodamine B, CMFDA or Ageladine A. The marine sponge alkaloid Ageladine A remained intracellularly trapped for several days in the worms, suggesting that it does not serve as substrate of multidrug resistance exporters. In addition, Ageladine A did not affect multidrug resistance-associated protein (MRP)-mediated dye export from *M. lignano* or the MRP1-mediated glutathione (GSH) export from cultured rat brain astrocytes. The data obtained demonstrate that life-imaging is a useful tool to address physiological drug export from intact marine transparent flatworms by using multiphoton scanning microscopy.

## 1. Introduction

The marine plathyhelminth *Macrostomum lignano*, presumably endemic to the Adriatic Sea, was described as a new species by Ladurner and colleagues in 2005 [1]. The flatworm inhabits shallow water sediments of the relative warm Adriatic Sea, where it experiences periods of hypoxia of variable length and subsequent reoxigenation, as well as possibly toxin exposure from its diatom diet. Additionally, in Adriatic shore sediments, the species may be exposed to different contaminants from anthropogenic sources. Thus, it is evident that *M. lignano* lives in a challenging environment, which requires organismic and cellular defense systems.

Secondary metabolites are a diverse group of organic compounds different from primary metabolites, they are not directly involved in development, growth, or reproduction but rather have allelochemical function. In some cases these functions are well defined within the competitive arms race or as feeding repellents, trail markers, sexual hormones, or antifouling substances (e.g., [2,3]). Some marine organisms are especially rich in secondary metabolites with important biological activity. Environmental stress such as predation, competition for space, and overgrowth is assumed to be the reason for the development of highly bioactive secondary metabolites for chemical defense in sponges [4]. The compound Ageladine A is a biological active brominated pyrrole-imidazole alkaloid, which was firstly isolated from the Papua New Guinean sponge *Agelas nakamurai* by Fujita and colleagues [5]. Three years after the first description of Ageladine A, Meketa and Weinreb [6] and Shengule and Karuso [7] developed a protocol for synthesizing the alkaloid which was further optimized [8,9]. Ageladine A shows intense fluorescence during UV-excitation [5,10], which was later demonstrated to be pH-dependent and applicable as a membrane permeable dye for staining of isolated cells and transparent organisms [10,11,12,13]. Several investigations of its bioactivity revealed an inhibitory effect on angiogenesis [5,14].

Cellular transport mechanisms, which export substances from the cellular interior, constitute an important defense line of cells and organisms against toxic compounds. Multidrug resistance transporters in eukaryotes belong to the superfamily of ABC-transporters, which are named after their most characteristic feature, the highly conserved ATP-binding cassette [15]. The major ABC-transporters involved in multidrug resistance in cancer cells are P-glycoprotein (MDR1, P-gp, ABCB1), multidrug resistance-associated proteins (MRPs, belonging to the ABCC sub-family), and the breast cancer resistance proteins (BRCP1, ABCG2). The most intensively investigated multidrug resistance transporter is the 170 kDa MDR1, which was first described as a surface phosphoglycoprotein in a hamster cell line [16]. MDR1 plays a major role in protecting cells against toxic agents, mainly in the liver, the kidney, and the brain [17,18]. Expressed in endothelial cells of the blood-brain-barrier, MDR1 is involved in preventing the permeation of drugs and toxic agents into the central nervous system [18]. MDR1 transports hydrophobic and amphipathic compounds, including cancer drugs like Colchicine, Vinblastine, and Paclitaxel [19]. The 190 kDa MRP1, the first described member of the multidrug resistance associated proteins (MRP) family, was originally cloned from a human multidrug resistance lung cancer line [17]. MRP1 mediates the transport of glutathione-, glucuronate-, and sulfate-conjugated as well as of unconjugated organic, anionic drugs and dyes [20]. Furthermore, MRP1 transports neutral as well as basic drugs. MRP2 and MRP3 also mediate the transport of glutathione conjugated toxins [21]. Although several isoforms of MRPs have been studied in the last decades, none have been associated as clearly with multidrug resistance as MRP1 [21,22].

Specific inhibitors for multidrug resistance transporters applicable in cancer patients, animals, and living cells are rare [21]. Verapamil was one of the first compounds to be identified as a not very specific inhibitor for P-glycoproteins [23]. Its presence enhances the accumulation of some anti-cancer drugs in MDR1-expressing model systems [24]. It is known that fluorescence dyes like Calcein am, Fluo-3 am, and Fura-2 am are actively extruded by MDR1 [25,26]. Therefore, they are well suited as reporter dyes to show the inhibitory effect of Verapamil on MDR1-mediated transport. MRPs are inhibited by Probenecid and Vinca alkaloids [27,28]. Gollapudi *et al.* showed that Probenecid reversed multidrug resistance in a cancer cell line by the modulation of the MRP-mediated drug transport [29]. MK571, a leukotriene D4-receptor antagonist, was shown to be a highly specific MRP1 inhibitor in clinical vesicular transport experiments [30]. By measuring the efflux of Calcein am, Fluo-3 am, and Fura-2 am, the inhibitory effect of Probenecid and MK517 can be determined since all three fluorescent dyes are actively transported by MRPs [31,32,33,34].

Human MRP and MDR homologues have been described in a wide variety of evolutionary early organisms, from plants, yeast, and different types of worms, including parasites. Sato *et al*. [35] demonstrated the presence of a MRP homologue in the protonephridial epithelium of the platythelminth *Schistosoma mansoni*. In aquatic animals, as well as in parasites, the whole body may be exposed to toxins or toxicants. The mechanisms of multidrug resistance in marine sponges, freshwater fish, and both freshwater and marine mussels are similar to multidrug resistance in human cancer cells [36]. Homologues of MDR1 and MRPs can be found for example in crustaceans [34,37], fish [38], mussels and diatoms [39,40,41].

Prior to this study, experiments were performed to estimate the pH values in tissues and cells of marine transparent animals (jellyfish, sea anemone, flatworm) by using the alkaloid Ageladine A as a pH sensitive fluorescence dye [10,11]. Remarkably, the fluorescent signal of Ageladine A was still detectable in the worms even several days post-staining. The question arose whether Ageladine A is fluorescing in the animal tissues longer than some other common fluorescent dyes, and if so, what could be the mechanism behind the persistent fluorescence. We hypothesized that Ageladine A fluorescence may be maintained by an inhibition of multidrug-resistance transporter-mediated export from the worms, possibly induced by the alkaloid itself. Therefore, we investigated the uptake and removal of reporter dyes under the influence of Ageladine A.

*M. lignano* is easy to culture, and due to its small size and transparency, it is a good candidate for optical investigations of living, undisturbed animals. By using multiphoton scanning microscopy, we have investigated whether homologues of MDR1 and MRPs are functionally expressed in the flatworm and whether Ageladine A may function as an inhibitor or substrate of such transporters. For the latter question, rat astrocytes were used as an established MRP1-expressing model [22] in a comparative approach. This is the first study addressing the presence, physiology, and substrate specificity of multidrug-resistance transporters in *M. lignano*.

## 2. Results

**Figure 1:**
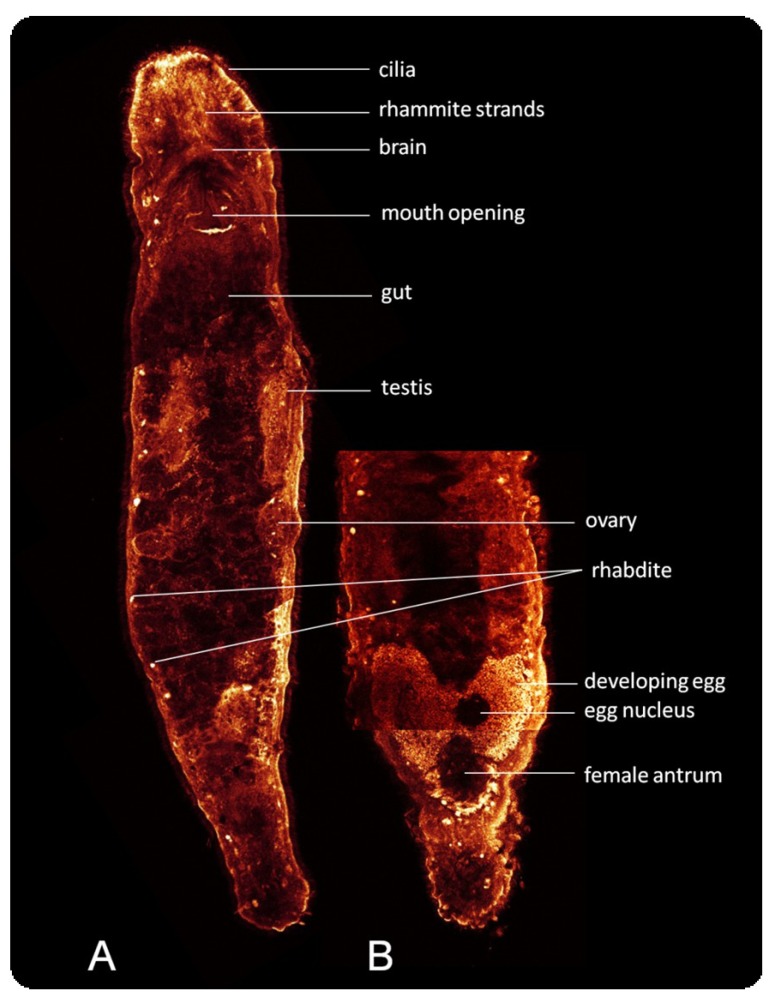
CFLSM scan of two individuals of *M. lignano* stained with Ageladine A (fluorescence is shown in false color). Ageladine A shows a pH-depending fluorescence. Highly fluorescent areas (white) are more acidic than dark areas (dark red). (**A**) Scan of the whole body. (**B**) Section of reproductive organs.

Ageladine A stains complete *M. lignano* individuals with the highest fluorescence coinciding with the rhabdites and some parts around the mouth (Figure 1). Due to the properties of Ageladine A, these areas are assumed to be the most acidic regions/organs [12]. Individual stainings with Ageladine A and Rhodamine B lasted more than 6 days after a short incubation period of roughly 2 h, whereas Fura-2 am and Fluo-3 am staining was maintained only during day 1.

**Figure 2:**
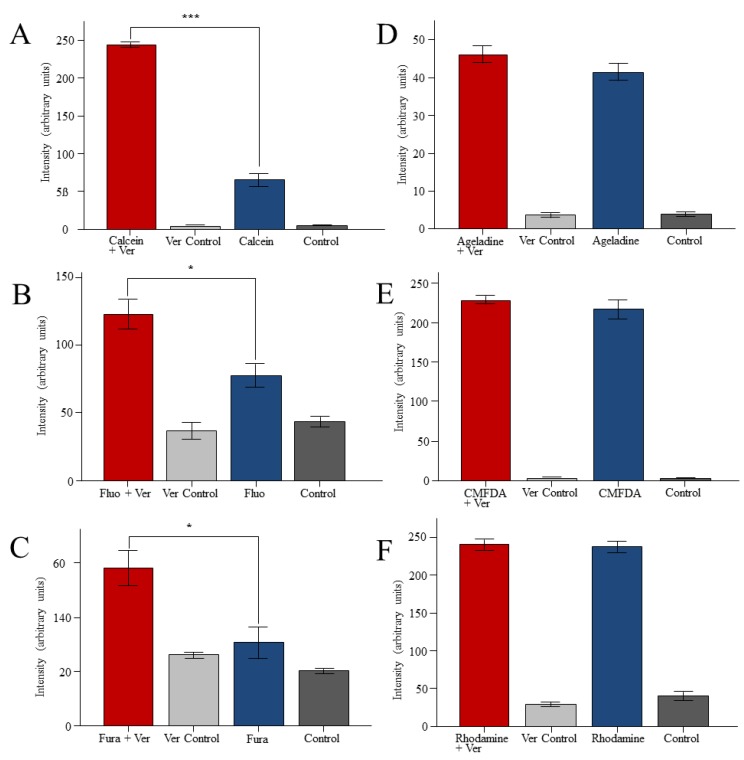
Effects of the MDR1-inhibitor Verapamil on the fluorescence of dye-treated *M. lignano*. The worms were exposed to the fluorescence dyes Calcein am (5 μM, **A**), Fluo-3 am (5 μM, **B**), Fura-2 am (5 μM, **C**), Ageladine A (5 μM, **D**), CMFDA (10 μM, **E**) or Rhodamine B (5 μM, **F**), in the absence or presence of Verapamil (Ver, 25 µM). * *p* < 0.05, ** *p* < 0.01, *** *p* < 0.001 (Student’s *t*-test).

We used three different dyes for the reporter assay: Calcein am, Fluo-3 am and Fura-2 am. Living worms were incubated with these dyes in the presence and absence of the MDR1 inhibitor Verapamil. Staining with all dyes was improved by Verapamil, indicating the involvement of a Verapamil-sensitive transporter in the export of these dyes (Figure 2A–C). Rhodamine B and CMFDA as well as Ageladine A stained the animals, but the addition of Verapamil did not affect the staining intensity (Figure 2D–F). This indicates that Ageladine A, Rhodamine B and CMFDA are not exported by MDR1 transporters.

**Figure 3:**
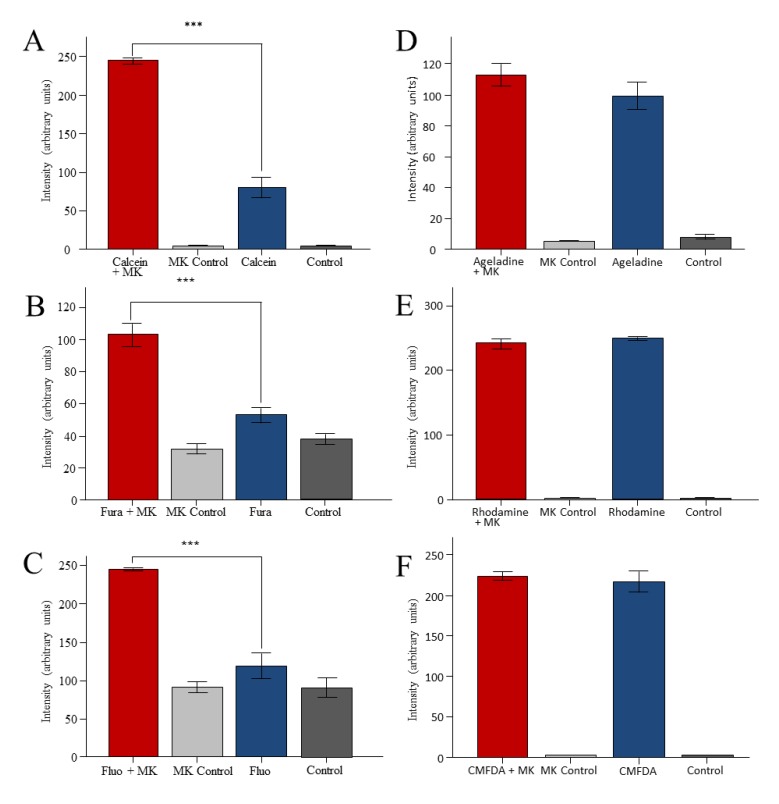
Effects of the MRP-inhibitor MK571 on the fluorescence of dye-treated *M. lignano*. The worms were exposed to the fluorescence dyes Calcein am (5 μM, **A**), Fura-2 am (5 μM, **B**), Fluo-3 am (5 μM, **C**), Ageladine A (5 μM, **D**), Rhodamine B (5 μM, **E**), or CMFDA (10 µM, **F**) in the absence or presence of MK571 (MK, 50 µM). *** *p* < 0.001.

Presence of the MRP1 transport inhibitor MK571 increased the fluorescence of Fura-2, Fluo-3, and Calcein (Figure 3A–C), indicating presence of a MK571 sensitive transporter in *M. lignano*. In contrast, MK571 did not increase the fluorescence of worms treated with Rhodamine B, Ageladine A or CMFDA (Figure 3D–F), suggesting that theses dyes are not substrates of a MRP-homologue in *M. lignano*. Similar results were obtained with the less specific MRP inhibitor Probenecid (data not shown).

**Figure 4:**
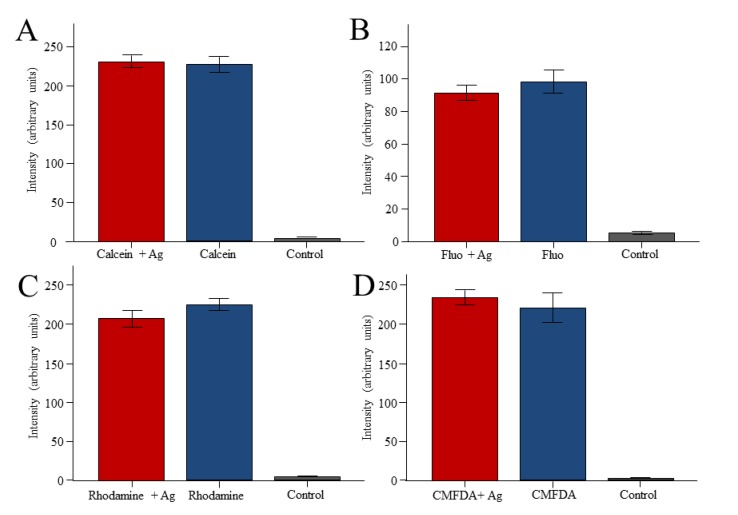
Effect of Ageladine A on the accumulation of fluorescent dyes in *M. lignano*. The worms were exposed to Calcein am (5 μM, **A**), Fluo-3am (5 μM, **B**), Rhodamine B (5 μM, **C**), or CMFDA (10 µM, **D**) in the absence or the presence of Ageladine A (Ag, 5 μM).

As Fluo-3 and Calcein are transported by MDR1 as well as by MRP transporters, we added Ageladine A to study a possible inhibitory effect of this compound on the transporters involved. However, Ageladine A had no effect on the accumulation of either fluorophore by the worms (Figure 4).

**Figure 5:**
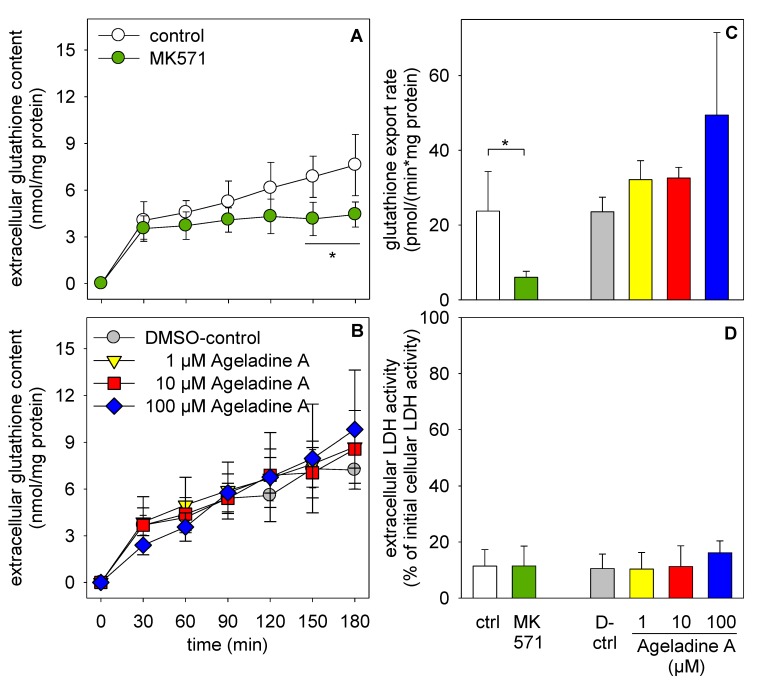
Effects of Ageladine A on the viability and on the GSH export from cultured astrocytes. The cells were incubated for up to 3 h in incubation buffer without (control) or with 50 µM MK571 (**A**) or without or with Ageladine A in the concentrations indicated (**B**). The export rates for glutathione (GSH) (**C**) were calculated from the almost linear increases in extracellular GSH content (A,B) between 30 and 180 min. The extracellular lactate dehydrogenase (LDH) activity was determined after 3 h of incubation (**D**). Statistical significance of the difference between data obtained from cells that had been treated without or MK571 (* *p* < 0.05) were calculated by the *t*-test. No significant differences (ANOVA with a Dunnet’s *post hoc* test) compared to control cells (D-control: 1% DMSO as solvent control) were found for Ageladine A-treated cells (*p* > 0.05). Experiments were performed on three (A–C) or two (D) individually prepared cultures.

In order to confirm the lack of effect of Ageladine A on MRP transporters, we used an established model to investigate the function of these transporters: the GSH export from cultured brain astrocytes [22]. Ageladine A was not toxic to astrocytes and did not significantly increase the extracellular lactate dehydrogenase activity, even in concentrations of up to 100 µM, demonstrating that the cell membrane integrity of the astrocytes was not compromised by Ageladine A (Figure 5D). MK571 clearly inhibited GSH transport from astrocytes (Figure 5A,C), whereas Ageladine A did not significantly inhibit GSH export (Figure 5B,C).

## 3. Discussion

The question we addressed was how *M. lignano* deals with exposure to toxins and toxicants. Using various fluorescent dyes as reporters with well-established pharmacologically inhibitors of multidrug transporters, we demonstrate the presence of functional multidrug resistance proteins in the worms that appear to have a pharmacological profile which resembles that of the mammalian transporters MDR1 and MRP1. These results demonstrate that the fluorescent dyes are taken up into the worms. We further ruled out the possible inhibition of multidrug resistance transporter-mediated dye export from *M. lignano* by the sponge derived secondary metabolite Ageladine A and showed that Ageladine A has no direct toxic or lytic effect on the worms for a minimum of a week.

The flatworms appear to have the ability to quickly and sustainably remove acetoxymethyl ester dyes as xenobiotic surrogates. In many experiments, staining of animals with such dyes was weak in the absence of inhibitors of the multidrug resistance transporters, indicating efficient constitutive export activity. Functional expression and a baseline activity support the idea of an evolutionary adaptation to toxin exposure from the diatoms contained in the diet and from additional uptake of anthropogenic pollutants at the Adriatic coast.

### 3.1. Multidrug Resistance Transporters

The structure and function of MDR1 and MRP1 are highly conserved in evolution [42,43,44,45,46,47,48,49]. An EST (expressed sequence tag) database for *M. lignano* [50] annotated the MDR (P-glycoprotein) sequence blasting against rodent genes [51]. Now, our data experimentally confirm the presence of functional multidrug resistance transporters in *M. lignano* that have a similar pharmacological profile as those known in mammals. Typical substrates of these transporters are hydrophobic or amphipatic compounds reaching 300–2000 Da in mass [52] fitting well to the substrates we have used in our study.

Seelig [53] compared a hundred compounds which were tested to be MDR1 substrates and described that two or three electron donor/hydrogen bonding acceptor groups are required for an interaction with MDR1. The spatial distance between the electron donor groups has to be either 2.5 ± 0.3 Å (Type I) or 4.6 ± 0.6 Å (Type II) to bind to the transmembrane sequence of MDR1 which contains many amino acids with hydrogen bonding donor side chains. The strength of the hydrogen bonds formed by substrate and transporter defines the dissociation of the transporter-substrate complex [54]. Therefore, a molecule having many hydrogen bonding sites operates as an inhibitor in most cases [54]. Furthermore, substrates containing planar aromatic rings and tertiary amino groups favor an interaction with MDR1 [55]. To understand why Ageladine A does not have an effect on multidrug resistance transporters and, furthermore, why it is not a substrate of such transporters, it is necessary to consider the molecular interaction between substrate and transporters. The ABC transporter function is directly linked to the cellular energy level which is a function of the rate of ATP hydrolysis to ADP and P_i_ [56].

**Figure 6:**
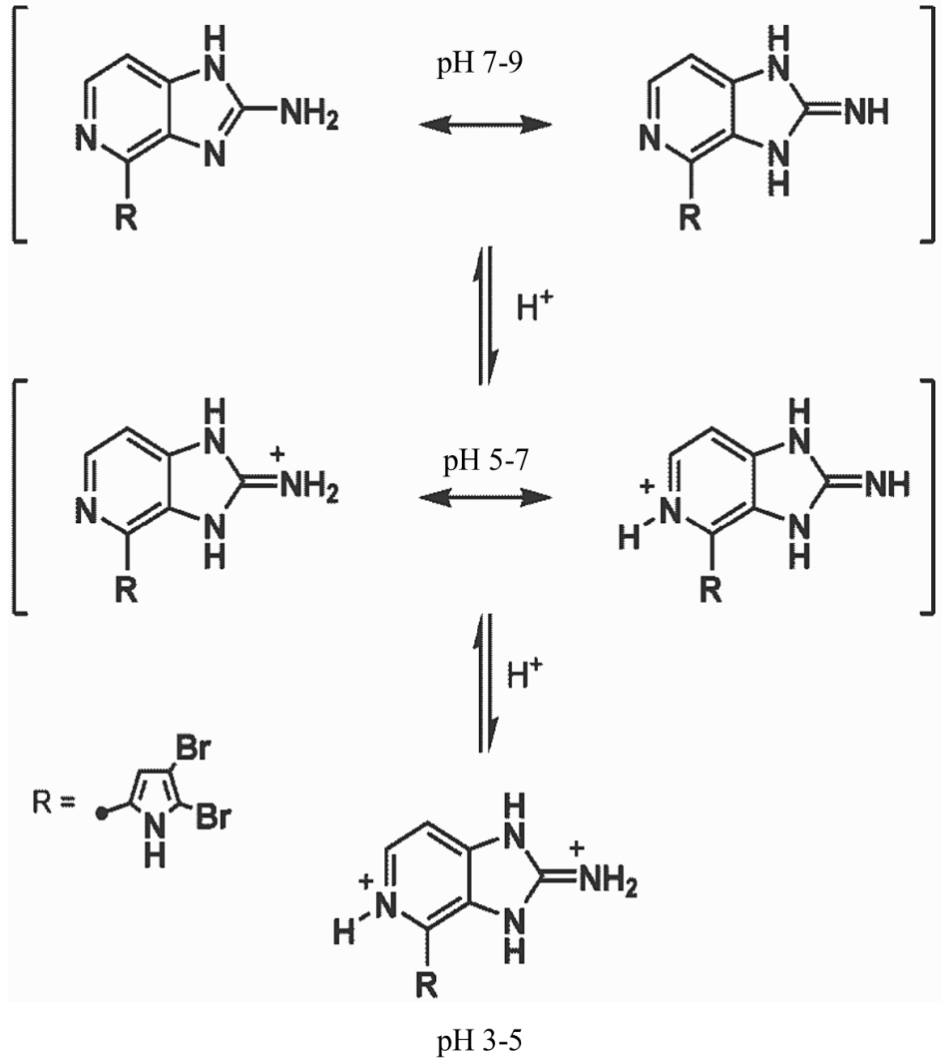
Mesomeric microspecies of Ageladine A. The distribution of the microspecies of Ageladine A depends strongly on the pH of the environment. The pyrrole is shown as residual structure (R). Two uncharged mesomeric microspecies are dominant at a pH of approximately 7–9. The single protonated microspecies are present at a pH of approximately 5–7, whereas the twice positively charged microspecies can be found at a pH of approximately 3–5 (Bickmeyer *et al*. 2010 (supplemental data in [11])).

Seawater and F/2 medium have a pH value around 8.1 and in this neutral to slightly basic environment Ageladine A is uncharged and hydrophobic, turning it into a membrane-permeable dye [11]. Inside acidic organelles, e.g., lysosomes, the molecule becomes charged (Figure 6). MDR1 is expected to recognize its substrates within the cell membrane before it reaches the cytoplasm to prevent the cellular entry of drugs [56,57]. This implies that only uncharged Ageladine A from a neutral or basic environment can theoretically be transported by MDR1. The charged microspecies of Ageladine A, “trapped” inside acid organelles, are not available for multidrug resistance transport in the cell membrane. This could explain the long lasting fluorescence of Ageladine A within the worms’ cells. Furthermore, Ageladine A features two electron donor groups (Figure 6) but their spatial distance seems to be too large to interact with the MDR1 hydrogen bonding donor side chains. The approximate distance between the nitrogen atom of the guanidine and the pyrrole ring (calculated at the B3LYP/6-311+G(d,p) level of theory [58]) is 585 pm, and this distance does not rank Ageladine A among either type I or type II substrate of Seeligs classification [53]. These considerations argue strongly against the protonated micro-species of Ageladine A to be a MDR1 substrate, which is in line with the results of our experiments.

The MRP transporters are described as organic anion transporters. Jedlitschky [59] and coworkers found that most of the anionic substrates are conjugates of glutathione, glucuronate, or sulfate. The highly charged substrates of MRP are not able to penetrate cell membranes [32,59]. For studying MRP in intact cells, hydrophobic precursors of the substrates are used which are converted intracellularly into anions [59]. As Ageladine A is an amphipathic molecule and tends to be neutral or cationic at the physiological pH of organic tissues, it does not meet the main requirement for being a MRP substrate, the anionic character. In contrast, a potentially formed conjugate of Ageladine A with glutathione, glucuronate, or sulfate could enable extrusion of the alkaloid from cells as such conjugates are well known as substrates of MRPs [60]. However, such a conjugation and export is unlikely to occur as the cellular fluorescence of Ageladine A is maintained over many days in the worms. Possibly the marine natural alkaloid compound Ageladine A was optimized in evolution to avoid export by multidrug resistance transporters. As control for a potential effect of Ageladine A on MRP-mediated transport, we investigated the GSH export from cultured brain astrocytes. Ageladine A did even in concentrations of up to 100 µM not compromise the viability of astrocytes which is consistent with the reported lack of Ageladine A toxicity on cultured neurons [11]. In contrast to MK571, Ageladine A did not inhibit MRP1-mediated export of GSH in astrocytes, which is consistent with the view that Ageladine A is not a substrate of MRP1.

Ageladine A does not seem to either reduce the ATPase activity or compete against normal substrates for an interaction with multidrug resistance transporters. Furthermore, our results indicate that Ageladine A cannot be considered as a catalytic substrate. The assays of our study only show the retention of the protonated fluorescent micro-species of Ageladine A. A possible extrusion of uncharged lipophilic micro-species of Ageladine A cannot be assessed through the use of this assay. Our results indicate that the protonated form of Ageladine A is a non-substrate of multidrug resistance transporters that interacts neither with the ATPase activity nor with the substrate binding domains.

The multidrug resistance transporter inhibitors used in our study were developed for medical purposes. MK571, for example, was discovered as a competitive leukotriene D4 receptor inhibitor in human tumor cells [30]. Probenecid was introduced in 1950 as “Benemid” and suggested as an adjunct to chemotherapy [61]. It was later used to study the effects of uric acid secretion and associated changes of blood levels in gout patients [62]. Furthermore, Verapamil was used to improve angina pectoris pain [63]. Thus, it is remarkable that all three inhibitors showed an effect in the flatworm *M. lignano*, which is not closely related to mammals. The study of Scherer *et al.* [41] showed a similar effect of Verapamil on transporters of the diatom *Thalassiosira rotula*. Our results support the fact that multidrug resistance transporters are highly conserved structures in nature.

### 3.2. Reporter Dyes

Sarkadi and Müller introduced Calcein am as “the dye of choice” for fluorescent reporter assays [64]. The hydrophobic non-fluorescent Calcein am is a substrate of MDR1, whereas the free hydrophilic form is trapped inside the cells and is no longer subject to transport. In MDR1 expressing cells, Calcein am is extruded before it can be intracellularly converted into its fluorescent form. Consequently, the Calcein accumulation is slow, unless a transport inhibitor is present. The free Calcein does not bind to proteins and is independent of changes in pH and ion concentrations [64]. Furthermore, it was shown that both Calcein am and free Calcein are transported by MRPs [32,33]. Fluo-3 am and Fura-2 am are further fluorescent anions which are used to determine MDR1 and MRP expression levels and to screen for modulators of the transporters [56].

Rhodamine B was additionally used as a reported dye. The literature is controversial about the function of Rhodamine B in MDR1- or MRP-mediated transport. Minier and Moore [65] suggest a correlation of Rhodamine B retention and expression levels of proteins which are immunorelated to MDR1 in mussel hemocytes. Furthermore, a Rhodamine-binding site was identified in MDR1 [66]. Several Rhodamine dyes, such as Rhodamine B, are transported by MDR1 and also stimulate the ATPase activity. According to our results, Rhodamine B does not seem to be either a MDR1 or a MRP substrate, because the presence of Verapamil, Probenecid, or MK571 inhibited the transporter function, but did not enhance Rhodamine B fluorescence. This is in line with results of previous studies [67] showing that Rhodamine B is either a very poor or non-substrate of MDR1 and MRP1. Structural and functional differences of transporters of different species may be responsible for controversial findings concerning Rhodamine B transport [68].

CMFDA is not mentioned to be a substrate of multidrug resistance transporters in the literature and is a good long-term cell tracker. The cell-impermeant S-conjugate does not leak out of cells [69]. In our experiments, the cellular fluorescence was not influenced by the presence of the multidrug resistance transporter inhibitors, suggesting that this dye is a non-substrate of multidrug resistance transporters in *M. lignano*.

## 4. Experimental Section

### 4.1. Animals and Cell Culture

The plathyhelminth *M. lignano* (DV-1 line) was cultured at 20 °C with 95% humidity and a 14/10 day-night cycle in glass dishes with nutrient-enriched artificial seawater (Guillard’s F2 medium) as previously described by Ladurner *et al.* [1]. Animals were fed once per week with the diatom *Nitzschia* sp.

Astroglia-rich primary cell cultures were obtained from the brains of neonatal Wistar rats. The cells were obtained as previously described [70] and seeded in 24 well plates at a density of 300,000 cells per well in 1 medium (90% DMEM, 10% fetal calf serum, 20 units/mL penicillin G, 20 μg/mL streptomycin sulfate). Medium was changed every week. Cells were maintained in a cell incubator (Sanyo, Osaka, Japan) in a humidified atmosphere containing 10% CO_2_. Triplicate experiments were performed on two-week-old cell cultures.

### 4.2. Chemicals

Six different fluorescent dyes were used. Ageladine A (exc: 365 nm, em: 420–480 nm) and the calcium indicators Calcein acetoxymethylester (Calcein am, Invitrogen; exc: 467 nm, em: 500–540 nm), Fura-2 acetoxymethylester (Fura-2 am, Sigma-Aldrich; exc: 360 nm, em: 480–540 nm), and Fluo-3 acetoxymethylester (Fluo-3 am, Invitrogen; exc: 488 nm, em: 510–550 nm) were used at a common concentration of 5 μM. All of the three calcium indicator dyes are known to be substrates of MDR1 [25,43,44] and MRPs [33,41,43,45].

Cell Tracker Green 5-chloromethylfluorescein diacetate (CMFDA, Invitrogen; exc: 488 nm, em: 500–550 nm concentration: 10 µM) and Rhodamine B (Sigma-Aldrich; exc: 561 nm, em: 570–620 nm; concentration: 5 µM) were also used.

MK571 (Biomol) was applied in a final concentration of 50 μM. It has been considered to be a specific inhibitor of MRP1 transporters [30]. Probenecid was used at a concentration of 2.5 mM, and Verapamil, one of the most potent competitive MDR1 inhibitors [46], was used at a final concentration of 25 µM.

### 4.3. Fluorometric Measurements

The fluorescence in *M. lignano* was monitored with a confocal laser scanning microscope Leica TCS SP5. The microscope was equipped with a multi-photon laser (MaiTai, DeepSee, Newport Spectra-Physics), an argon-laser (488 nm), and a helium-neon-laser (561 nm). For analyzing differences in fluorescence, the region-of-interest function of the software Leica LAS AF was used, with five transects drawn through each animal’s body covering all tissues.

### 4.4. Stability of Staining

The fluorescence intensity of six dyes was investigated for up to nine days in stained *M. lignano*. The experimental animals were transferred to Petri dishes containing fresh F/2 medium (without food) to avoid fluorescence interference from diatoms, *etc*. Animals were placed on slides filled with 100 μL F/2 medium containing the respective fluorescent dye. After 1.5 h of incubation, animals were transferred to fresh F/2 medium without fluorescent dyes. To avoid contamination and evaporation of the medium, slides were kept in plastic boxes sealed with foil. To measure the fluorescence intensity animals were transferred into Utermöhl chambers containing 100 μL F/2 medium with 5 µL of a relaxing solution (2 g MgCl_2_ in 1 mL filtered artificial seawater) to facilitate the scanning.

### 4.5. Inhibition of Multidrug Resistance Transporters

The MDR1-inhibitor Verapamil and the MRP1 inhibitors MK571 or Probenecid were added to the F/2 medium containing one of the listed fluorescent dyes (Ageladine A, Calcein am, Fura-2 am, Fluo-3 am, CMFDA or Rhodamine B). An inhibition of the transporters should cause a stronger accumulation of fluorescence substrates in the worms by inhibiting dye export. Changes in fluorescence intensity were measured with a Leica SP5 confocal microscope after 1.5 h of incubation. Furthermore, Ageladine A was added to the medium containing fluorescent dyes to test for transporter inhibition of this compound. Ageladine A and Fura-2 am have overlapping excitation and emission ranges, which could lead to fluorescence interference among dyes. Therefore, combined staining of Fura-2 together with Ageladine A was avoided.

### 4.6. Determination of Glutathione Content and Viability of Treated Astrocytes

The extracellular amount of glutathione (GSH; determined as GSx (=amount of GSH + 2 × amount of glutathione disulfide)) was analyzed to test for a potential effect of Ageladine A on MRP1-mediated GSH export. Astrocytes were incubated in incubation buffer without (control) or with 1% DMSO (solvent control), 50 μM MK571, 1 μM, 10 μM, or 100 μM Ageladine A for up to 3 h as previously described and extracellular GSH was determined as described previously [71] in microtiter plates, according to the method originally described by Tietze [70]. Cell viability was determined as previously described [22] by determining the extracellular lactate dehydrogenase (LDH) activity after incubation and comparing it with that of astrocytes that had been totally lysed by a 30 min incubation in 1% Triton X-100. The protein content of the cells was determined by the Lowry methods [72] using bovine serum albumin as standard protein.

### 4.7. Statistics

Fluorescence intensities from five regions of interest per animal were pooled, averaged, and considered as one value. Each treatment group consisted of 5–10 individuals. Results are presented as means ± SE. Data generated by lactate dehydrogenase and GSH assays measurements represent means ± SD of three independent experiments. General data processing was conducted with Microsoft Excel 2010. The statistical tests ANOVA and nonparametric *T*-tests were performed, using the software IBM SPSS 20.

## 5. Conclusions

The presented work is a multidrug resistance study on the living flatworms *M. lignano,* and provides evidence for the presence of at least two classes of highly evolutionary conserved multidrug resistance transporters that have the pharmacological profile of MDR1 and MRP1. The results shown disprove our original hypothesis that Ageladine A may have an inhibitory effect on MDR1- and/or MRP-mediated export processes. Nevertheless, the lack of export of Ageladine A together with the apparent low toxicity of this compound make Ageladine A a highly useful tool for life imaging of marine transparent animals and cells.
